# The Worst War of the World

**Published:** 1917-07-28

**Authors:** 


### THE WORST WAR OF THE WORLD.

### I. ITS TEACHINGS AND MOMENTOUS ISSUES.

Three years of war, forced upon the British Nation and Empire with its Allies, the objective of the aggressors being the destruction or complete mastery of kindred nations, whom they attacked without warning, in the hope of reducing Europe to a condition of dependence, involving slavery, outrage, injustice and wrong, have stirred the heart-strings of all free peoples, and united them in defence of the freedom of the world and the protection of the weak. So complete is the unity of our nation that it has given us a dominant influence in the national policy of the Allies, and an attractive force which has aroused the whole Anglo-Saxon race and joined them in the fight for freedom and righteousness. History contains nothing more remarkable than this unity of the nations, resting upon the highest principles of righteous determination to see wrong righted, and freedom maintained, everywhere, amongst the peoples of the world on democratic bases. From time to time we have insisted upon the paramount importance of every man and woman realising their spiritual as well as their physical nature. The war has quickened the sense of the spiritual nature of man in the majority of individuals, united them in the succour of the helpless and wronged, motives which rest upon principles which Christ himself upheld, taught, and died to maintain and enforce. In consequence, the views and habits of our people have changed, they are quickened with the desire to realise the highest aspirations which men and women can attain to. Nothing more striking has been seen than the overwhelming support of a Government which has swept aside party issues, and made them, and very many other things, subordinate to a policy which insists upon getting on with the war, and so conducting it as to secure the triumph of principles which rest upon righteousness. They stand indeed, for the salvation of humanity from the forces of evil, tyranny, self-seeking, robbery, crime in its worst and most manifold form, and the degraded ambition of men in authority, to rule the universe by a system of brute force, resting upon militarism in its most life-destroying and hateful form. Small wonder, then, that when the people of Great Britain and the Empire realised what the present war meant, they should resolve, with dogged determination, to bend all their energies to put an end to the German system of brute force, and to render it impossible that it and militarism should survive the termination of the war. They felt that such a system must never be permitted to raise its head again with success, for the destruction of freedom, and the enforcement of principles of government, that eliminate the spirit of justice and fair-dealing between man and man, without which the world would become impossible for the majority of men and women, who believe in eternal justice, and the enforcement of righteousness in the government of its affairs.

The history of wars in the past does not encourage the seeker after knowledge, to expect any remarkable exhibition of high principle or self-sacrifice on the part of workers, who devote their whole energies to the supply of munitions of war, and of the necessary implements, the primary object of which is to destroy life, and annihilate millions of men in the course of a great war. But the war British people with their Allies are waging at the moment is unlike any conflict between nations which the world has had to endure in the past, because the peoples of the majority of the European and other nations are united, in opposing the dominance of a tyranny, resting upon militarism, which, if permitted, would certainly destroy the Allies and their people did they not now unite and put forth all their strength to resist and overthrow it. Indeed, the task is so tremendous, that it can only be accomplished, under God's good providence, by the whole-hearted, self-denying efforts of each and all the peoples, who collectively constitute the Allied nations. Hence the war worker in the present war, as we hope to show, includes both sexes, practically the whole civilian population, as well as the men who are bravely facing the heat and dangers of battle on the seas and on land, that the inhabitants of Europe and all the world may be free to live their lives, and to govern themselves in accordance with the wishes of the majority of the people concerned, whom freedom has endowed with the spirit of independence, and a paramount realisation of their right to manage their own affairs, and to conduct the business of each nation, under a system of government, resting upon approved lines best calculated to secure the greatest good of the greatest number.

At the outset of the war no nation except the aggressors was ready or in a condition to enter upon a great war at all. Indeed the main assurance of their ultimate triumph, which animated the Germans and their rulers, was the feeling, that their forty years' preparation for war was upon so gigantic a scale that it would enable them, within a few weeks or months, to conquer Europe, and to enforce upon the world the will of the militarist party. Being themselves mastered by this party largely through tyranny and brute force, the Germans had formed the ambition to keep neighbouring peoples under their control, and to enforce their will, everywhere, by means which the war has proved to be in fact, the most cruel, murderous, brutal, cowardly, man-slaying, and woman-debasing system, that the powers of evil have yet found it possible to elaborate for the destruction and annihilation of everything that is best in humanity, and the elimination of the noblest types of both men and women. Small wonder, then, that the practically unanimous feeling among the Allied Nations is, "What can I do to help forward the war and free the people from the evil consequences to humanity which must follow the triumph of Germany and its rulers?"

*(To be continued.)*

